# Supplementary material for: Pyruvate Dehydrogenase Kinase 1 inhibition mediated oxidative phosphorylation enhancement in cartilage promotes osteoarthritis progression
Source: BMC Musculoskelet Disord. 2023 Jul 20;24:597. doi: 10.1186/s12891-023-06585-6 (PMC10357736; doi:10.1186/s12891-023-06585-6)
Supplement: Supplementary file 1 — Additional file 1. [file 12891_2023_6585_MOESM1_ESM.zip › BP.Gsea.1653623667859/GOBP_PLACENTA_BLOOD_VESSEL_DEVELOPMENT.html]

Details for gene set GOBP\_PLACENTA\_BLOOD\_VESSEL\_DEVELOPMENT[GSEA]

|  || Dataset | 55457\_collapsed\_to\_symbols.cls.cls #OA\_versus\_normal.cls.cls #OA\_versus\_normal\_repos |
| Phenotype | cls.cls#OA\_versus\_normal\_repos |
| Upregulated in class | normal |
| GeneSet | GOBP\_PLACENTA\_BLOOD\_VESSEL\_DEVELOPMENT |
| Enrichment Score (ES) | -0.5140623 |
| Normalized Enrichment Score (NES) | -1.738756 |
| Nominal p-value | 0.006504065 |
| FDR q-value | 0.12038552 |
| FWER p-Value | 1.0 |
Table: GSEA Results Summary

  

Fig 1: Enrichment plot: GOBP\_PLACENTA\_BLOOD\_VESSEL\_DEVELOPMENT      
 Profile of the Running ES Score & Positions of GeneSet Members on the Rank Ordered List

  

| SYMBOL | TITLE | RANK IN GENE LIST | RANK METRIC SCORE | RUNNING ES | CORE ENRICHMENT || 1 | AKT1 | AKT serine/threonine kinase 1 [Source:HGNC Symbol;Acc:HGNC:391] | 1192 | 0.478 | -0.0407 | No |
| 2 | WNT2 | Wnt family member 2 [Source:HGNC Symbol;Acc:HGNC:12780] | 2700 | 0.279 | -0.1274 | No |
| 3 | SPINT1 | "serine peptidase inhibitor, Kunitz type 1 [Source:HGNC Symbol;Acc:HGNC:11246]" | 3938 | 0.161 | -0.2060 | No |
| 4 | HEY2 | hes related family bHLH transcription factor with YRPW motif 2 [Source:HGNC Symbol;Acc:HGNC:4881] | 4058 | 0.150 | -0.1989 | No |
| 5 | VASH2 | vasohibin 2 [Source:HGNC Symbol;Acc:HGNC:25723] | 4345 | 0.124 | -0.2076 | No |
| 6 | OVOL2 | ovo like zinc finger 2 [Source:HGNC Symbol;Acc:HGNC:15804] | 5017 | 0.071 | -0.2520 | No |
| 7 | FZD5 | frizzled class receptor 5 [Source:HGNC Symbol;Acc:HGNC:4043] | 5392 | 0.041 | -0.2766 | No |
| 8 | TMED2 | transmembrane p24 trafficking protein 2 [Source:HGNC Symbol;Acc:HGNC:16996] | 5709 | 0.015 | -0.2995 | No |
| 9 | PKD2 | "polycystin 2, transient receptor potential cation channel [Source:HGNC Symbol;Acc:HGNC:9009]" | 6254 | -0.024 | -0.3392 | No |
| 10 | GGNBP2 | gametogenetin binding protein 2 [Source:HGNC Symbol;Acc:HGNC:19357] | 6301 | -0.027 | -0.3398 | No |
| 11 | MAPK1 | mitogen-activated protein kinase 1 [Source:HGNC Symbol;Acc:HGNC:6871] | 6853 | -0.068 | -0.3752 | No |
| 12 | HEY1 | hes related family bHLH transcription factor with YRPW motif 1 [Source:HGNC Symbol;Acc:HGNC:4880] | 8213 | -0.172 | -0.4621 | No |
| 13 | VASH1 | vasohibin 1 [Source:HGNC Symbol;Acc:HGNC:19964] | 8473 | -0.191 | -0.4614 | No |
| 14 | PKD1 | "polycystin 1, transient receptor potential channel interacting [Source:HGNC Symbol;Acc:HGNC:9008]" | 8755 | -0.213 | -0.4602 | No |
| 15 | RBPJ | recombination signal binding protein for immunoglobulin kappa J region [Source:HGNC Symbol;Acc:HGNC:5724] | 8800 | -0.217 | -0.4401 | No |
| 16 | ITGB8 | integrin subunit beta 8 [Source:HGNC Symbol;Acc:HGNC:6163] | 9442 | -0.268 | -0.4608 | No |
| 17 | NR2F2 | nuclear receptor subfamily 2 group F member 2 [Source:HGNC Symbol;Acc:HGNC:7976] | 10129 | -0.334 | -0.4778 | Yes |
| 18 | MAP2K1 | mitogen-activated protein kinase kinase 1 [Source:HGNC Symbol;Acc:HGNC:6840] | 10197 | -0.340 | -0.4461 | Yes |
| 19 | RBM15 | RNA binding motif protein 15 [Source:HGNC Symbol;Acc:HGNC:14959] | 10499 | -0.373 | -0.4290 | Yes |
| 20 | NOTCH2 | notch receptor 2 [Source:HGNC Symbol;Acc:HGNC:7882] | 10720 | -0.395 | -0.4032 | Yes |
| 21 | PLG | plasminogen [Source:HGNC Symbol;Acc:HGNC:9071] | 10939 | -0.423 | -0.3741 | Yes |
| 22 | FOSL1 | "FOS like 1, AP-1 transcription factor subunit [Source:HGNC Symbol;Acc:HGNC:13718]" | 11222 | -0.460 | -0.3461 | Yes |
| 23 | NSDHL | NAD(P) dependent steroid dehydrogenase-like [Source:HGNC Symbol;Acc:HGNC:13398] | 11670 | -0.527 | -0.3236 | Yes |
| 24 | HS6ST1 | heparan sulfate 6-O-sulfotransferase 1 [Source:HGNC Symbol;Acc:HGNC:5201] | 11932 | -0.579 | -0.2810 | Yes |
| 25 | SOCS3 | suppressor of cytokine signaling 3 [Source:HGNC Symbol;Acc:HGNC:19391] | 12453 | -0.725 | -0.2426 | Yes |
| 26 | JUNB | "JunB proto-oncogene, AP-1 transcription factor subunit [Source:HGNC Symbol;Acc:HGNC:6205]" | 12468 | -0.734 | -0.1641 | Yes |
| 27 | HES1 | hes family bHLH transcription factor 1 [Source:HGNC Symbol;Acc:HGNC:5192] | 12676 | -0.849 | -0.0880 | Yes |
| 28 | CCN1 | cellular communication network factor 1 [Source:HGNC Symbol;Acc:HGNC:2654] | 12802 | -0.970 | 0.0076 | Yes |
Table: GSEA details [plain text format]

  

Fig 2: GOBP\_PLACENTA\_BLOOD\_VESSEL\_DEVELOPMENT      
 Blue-Pink O' Gram in the Space of the Analyzed GeneSet

  

Fig 3: GOBP\_PLACENTA\_BLOOD\_VESSEL\_DEVELOPMENT: Random ES distribution      
 Gene set null distribution of ES for **GOBP\_PLACENTA\_BLOOD\_VESSEL\_DEVELOPMENT**
